# Healthcare Utilisation and Clinical Outcomes in Older Cardiovascular Patients Receiving Comprehensive Medication Management Services: A Nonrandomised Clinical Study

**DOI:** 10.3390/ijerph19052781

**Published:** 2022-02-27

**Authors:** Andrea Brajković, Lorena Bosnar, Mariana Martins Gonzaga do Nascimento, Ingrid Prkačin, Antonija Balenović, Djenane Ramalho de Oliveira, Iva Mucalo

**Affiliations:** 1 Faculty of Pharmacy and Biochemistry, University of Zagreb, 10000 Zagreb, Croatia; abrajkovic@pharma.hr; 2 Health Care Centre Zagreb–Centre, 10000 Zagreb, Croatia; lorena.bosnar@gmail.com; 3 College of Pharmacy, Centre for Pharmaceutical Care Studies, Federal University of Minas Gerais, Belo Horizonte, Belo Horizonte 31270-901, Brazil; marianamgn@yahoo.com.br (M.M.G.d.N.); djenane.oliveira@gmail.com (D.R.d.O.); 4 Department of Internal Medicine, School of Medicine, University of Zagreb, Merkur University Hospital, 10000 Zagreb, Croatia; ingrid.prkacin@gmail.com; 5 The Faculty of Medicine, University of Rijeka, 51000 Rijeka, Croatia; abalenovic@libertas.hr

**Keywords:** medication management services, nonrandomised, primary care, cardiovascular, older patients

## Abstract

The objective of this study was to evaluate the impact of comprehensive medication management (CMM) services on healthcare utilisation and cardiovascular risk factors among older patients with established cardiovascular diseases (CVDs). This quasi-experimental study that was performed at the Croatian primary care ambulatory clinic included patients aged 65 to 80 years. Patients were divided into intervention (65 patients) and control groups (68 patients) and were followed-up for one year. Pharmacists provided face-to-face consultations to patients from the intervention group. Groups were compared with regards to the clinical parameters (blood pressure, HbA1c, LDL, TC) and healthcare utilisation (hospital admission, emergency visits, unplanned GP visits). The CMM intervention significantly improved systolic blood pressure (*p* = 0.038), diastolic blood pressure (*p* = 0.001), total cholesterol (*p* = 0.014), low-density lipoprotein cholesterol (*p* = 0.005), and glycosylated haemoglobin (*p* = 0.045) in comparison with the control group. Patients included in CMM services had statistically and clinically lower systolic (−9.02 mmHg, *p* < 0.001) and diastolic blood pressure (−4.99 mmHg, *p* < 0.001) at the end of the study. The number of hospital admissions and unplanned GPs visits were 3.35 (95% CI 1.16–10.00) and 2.34 (95% CI 1.52–3.57) times higher in the control group compared to the intervention group, respectively. This study demonstrated that pharmacists providing CMM services can significantly contribute to better clinical outcomes and lower healthcare utilisation, thus potentially contributing to total healthcare savings.

## 1. Introduction

Cardiovascular diseases (CVDs) are the number one cause of global mortality, responsible for an estimated 17.9 million deaths each year [1]. Likewise, CVDs are the leading cause of death in Croatia, which, compared to other European countries, has a much higher death rate from diseases of the circulatory system than the European Union averages [2,3]. Furthermore, an ample worldwide evidence base suggests that patients with established CVDs are often inadequately treated or not offered therapies that are likely to bring them benefits [4,5]. Treatment of CVDs and their modifiable risk factors requires the use of multiple medications, thus predisposing patients to a higher risk of experiencing drug therapy problems (DTP) [6,7,8]. Therefore, in order for the effective and safe use of medications to be ensured, there is a call for actions aimed at strengthening primary healthcare services focused on medication management.

In the last few decades, pharmacists have played a crucial role in the medication management through the provision of various pharmaceutical services. However, comprehensive medication management (CMM) services are the only patient-centred pharmaceutical services supported by a vast amount of evidence-based literature in the scientific and clinical area [9,10] and are promoted by several organisations such as the American College of Clinical Pharmacy [11,12], Get the Medications Right Institute [13], and the Patient-Centered Primary Care Collaborative [14]. In this service, the fundamental purpose of the pharmacist’s work is to address all of a patient’s medication-related needs, optimise their medication use, and improve their health outcomes. In addition, pharmacists’ patient care process in CMM is made specific by a unique assessment process and a taxonomy that the professional applies to define the patient’s medication-related needs, both of which are embedded in the Medication Therapy Problem Framework adopted and promoted as a standard of practice by the Pharmacy Quality Alliance organisation [15].

Although the service has been established and reproduced in many countries worldwide, mainly the Anglo-Saxon countries [11,16,17], limited published data on CMM service implementation [18] confirms that the service has not been developed nor recognised in Europe. Croatia is one of the first European countries where the implementation of CMM services started, and this has occurred only recently through the pilot project at the primary healthcare site [18].

The benefits of CMM services are numerous and include better care [9,10,19,20,21,22,23,24,25,26], cost reduction [10,27,28], and improved patient and provider experience [10,29,30]. Thus far, various studies have demonstrated the positive impact of pharmacists’ interventions on the management of chronic diseases by improving individual cardiovascular (CV) risk factors such as blood pressure [22,24,31,32,33], glycated haemoglobin (HbA1c) [19,20,23,24,26,34], and LDL cholesterol [10,19,20,23,31], as well as on the reduction of patients’ utilisation of healthcare services [10,28,31,32]. However, to the best of our knowledge, the effect of the CMM services on healthcare utilisation and clinical parameters has not yet been evaluated among older patients with established CVDs at the primary care level. Hence, the aim of our study was to evaluate the clinical impact of CMM services on healthcare utilisation (unplanned office visits, emergency department visits, and hospitalisations) and CV risk factors (hypertension, glycated haemoglobin, lipid profile) among older patients with established CVDs in a primary public healthcare system.

## 2. Materials and Methods

### 2.1. Study Design and Setting

A prospective, open controlled pre- and post-intervention study was carried out from January 2018 to December 2020 at the primary care ambulatory clinic, Health Care Centre Zagreb–Centre (HCZC). HCZC is the largest county healthcare centre in Croatia, with 101 active general practitioner (GP) teams, and is the only healthcare centre providing CMM services in Croatia thus far. The HCZC’s CMM services, developed in partnership with the University of Zagreb (UoZ) Faculty of Pharmacy and Biochemistry, are provided by two pharmacists from the UoZ Faculty of Pharmacy and Biochemistry who facilitated the implementation of the CMM services at the health centre. The full implementation process of this new practice management system of CMM services was described elsewhere [18].

All Croatian citizens and residents have the right to healthcare through the compulsory mandatory health insurance scheme that provides universal health insurance coverage to the whole population. In Croatia, primary care physicians (GPs, paediatricians, and gynaecologists) are usually patients’ first point of contact with the health system, and each insured citizen is required to register with a GP (adults) or a paediatrician (children), whom they can choose freely [35]. There are not many group practices and interdisciplinary teams in primary healthcare in Croatia, and thus the inclusion of pharmacists as healthcare providers at the primary care level was an innovative and unique endeavour in the studied setting.

### 2.2. The Patient Care Process

Pharmacist practitioners providing CMM services followed the philosophy and the standardised patient care process, as proposed by Cipolle et al. [36]. Since all patient care providers need a structured, rational thought process for sound clinical decision retrieval, the Pharmacotherapy Workup developed as a systematic problem-solving process specific to the practice of pharmaceutical care was employed in this study. This validated standardised process is used to identify, resolve, and prevent DTPs; establish therapy goals; select interventions; and evaluate outcomes in order to achieve the better possible health results. Patients’ DTPs identified and addressed by CMM pharmacists were grouped into seven categories and always assessed in the same systematic order—first, the appropriateness of the drug therapy; followed by the effectiveness of the drug regimen; safety; and, at the end, adherence [36].

### 2.3. Sample Definition and Data Collection

In quasi-experimental designs such as an open controlled pre- and post-intervention research employed in our study, at least two separate groups are evaluated: one which receives the intervention of interest (CMM services); and another one that serves as a control or comparison group (usual care provided by GPs). Thus, the non-random control group is similar in design to a randomised controlled trial, except that patients are assigned to treatment groups in a non-random fashion. It should be emphasised that this type of quasi-experimental design is strongly supported by the World Health Organisation, as it enables researchers to use real-world processes and data [37].

#### 2.3.1. Study Subjects and Sample Size

The patients who were eligible for inclusion in our study (1) were aged 65 to 80 years, (2) had hypertension and at least one additional established CVD, and (3) were willing and able to sign an informed consent form. Patients with mental and behavioural disorders due to psychoactive substance use, with behavioural syndromes associated with physiological disturbances and physical factors, with cognitive impairment, or who were not able to decide independently on health-related aspects were excluded from the study. The sample size was calculated to detect a minimum difference of 7.5 mmHg between the groups, with a statistical power of 80% and a significance level of 0.05. A target sample size of 70 patients in each group was assumed to ensure statistical power and account for 20% dropouts during the study.

#### 2.3.2. Control Group

Patients included in the control group received the usual care, which included GPs and other specialists’ visits, on an “as-needed” basis. Their data were collected by the “control” GP, parallel with the collection of data for the intervention group. The control GP was not involved in the care of patients pertaining to the intervention group. Routine procedures administered to patients were recorded in the patient records and consisted of adjustments in prescribed therapy, requests for laboratory exams, general information about patient health, and specialist referrals.

#### 2.3.3. Intervention Group

In addition to the usual care provided by GPs and other healthcare providers, patients from the intervention group also received pharmaceutical care intervention (CMM services). On the basis of the pre-defined inclusion criteria, GPs and/or medical specialists identified patients and referred them to pharmacists. Moreover, self-referral by the patients was enabled. CMM services were provided through face-to-face consultations at the private counselling area, namely a pharmacotherapy counselling service located at the HCZC, and, when necessary by telephone, especially amidst the COVID-19 lockdown (a 4 month period in 2020).

The initial assessment was performed at the first consultation, followed by the provision of the care plan created in agreement with the patient and the GP. For the purposes of this study, “initial assessment” was defined as the first and second consultation to ensure that the pharmacist had been able to capture and evaluate all the health problems and medications used by the patient. On each following visit, follow-up consultations were conducted, and the frequency of follow-up consultations depended on the complexity of the drug therapy used by the patient and the number of DTPs identified by the pharmacist. The initial assessment lasted 60–90 min, and every follow-up encounter was 30–60 min. A minimum of 3 consultations were held for each patient. Communication with GPs took place in written form (electronic consultation system Health net. PRO; e-mail) and, if needed, by face-to-face or phone conversation. All the GPs included in the study had less than 10 years of professional experience in primary healthcare.

#### 2.3.4. Data Collection

All of the patients’ data, including sociodemographic data (gender, age, level of education and habits), anthropometric data (height, body weight, and body mass index), medical history (current and past medical conditions), utilised medications (prescription medications for chronic conditions, over-the-counter (OTC) medications, herbal remedies, supplements and medications used for a limited time), past medication use, allergies, and adverse drug events were collected during the initial assessment by a review of patients’ medical records, as well as through the interview with the patients. The International Classification of Diseases (ICD-10 Version: 2019) and Anatomical Therapeutic Chemical (ATC) Classification codes were used to analyse the principal diagnosis and comorbidities, and the drug therapy, respectively.

Clinical parameters such as systolic blood pressure (SBP, in mmHg), diastolic blood pressure (DBP, in mmHg), heart rate (HR, in bpm), low-density lipoprotein cholesterol (LDL-C, in mmol/L), triglycerides (in mmol/L), high-density lipoprotein cholesterol (HDL-C, in mmol/L), total cholesterol (TC, in mmol/L), glycosylated haemoglobin (HbA1c, in percentage), fasting blood glucose (FBG, in mmol/L), number of hospital admissions, number of emergency department visits, and number of unplanned GP visits were assessed at the baseline and following a 12 month period, for both groups. Number and types of DTPs, types of interventions implemented to resolve them, and changes in patients’ clinical status were collected during every follow-up consultation in the intervention group, along with the number of CMM consultations. During each encounter, patients’ data were thoroughly documented in the CMM documentation system.

### 2.4. Clinical Outcomes

The primary outcome was the difference in healthcare utilisation events between the two studied groups (hospital admission, emergency department visits, and unplanned GPs visits). Furthermore, within- and between-treatment differences in SBP; DBP; and serum levels of HbA1c, TC, LDL-C, HDL-C, and triglycerides were also calculated as primary outcome measures.

### 2.5. Data Analysis

The impact of CMM services on clinical outcomes was determined by measuring the differences between the intervention and the control group, and the differences between serial measurements within the same group with regards to the evaluated parameters. The total number and type of identified and resolved DTPs and clinical outcomes status were established by comparing the baseline values collected during the initial assessment with the 12 month follow-up end-point values only for the intervention group. During the initial stage of care plan development, the following parameters were established for each of the patients’ medical conditions and utilised in the evaluation of achieved therapy goals: SBP 130–139 mmHg, DBP 70–79 mmHg [38]; LDL-C < 1.8 (high CV risk); < 1.4 mmol/L (very high CV risk) [39]; improvement of clinical symptoms.

Quantitative variables were described according to their mean, standard deviation, median, and inter-quartile range, while categorical variables were shown as frequency and percentage. The Kolmogorov–Smirnov test was utilised to test the normality of the data distribution. Pearson’s chi-squared test was used to test the difference in baseline characteristics, as well as to compare the number of healthcare utilisation events between the groups. To compare the differences between baseline and end-point values within one group, we used a paired *t*-test. Factorial two-way ANOVA and Fisher’s LSD test were used to compare the differences between the baseline and end-point values between the intervention and the control group. The data were analysed with the STATISTICA, software, version 6.1 (StatSoft Inc, USA). A value of *p* < 0.05 was considered to be statistically significant.

## 3. Results

A total of 137 patients were included in the study, of which 69 patients pertained to the intervention group and 68 patients to the control group. Dropouts in the intervention group were four in number; one dropout was caused by death and three by loss of interest for further participation in the study. Analysis of baseline parameters revealed that the two groups were similar in all demographic and clinical parameters (*p* > 0.05). Overall, 133 participants (48 men and 85 women), aged 72.7 ± 4.7 years (mean ± SD), with essential hypertension and at least one established CVDs, and who met the eligibility criteria completed the study.

Cardiovascular medications were the most frequently prescribed group of medications (44.1%), followed by medications for alimentary tract and metabolism (18.0%), and nervous system medications (12.4%). Accordingly, diseases of the circulatory system were the most prevalent conditions (34.9%), followed by endocrine, nutritional, and metabolic diseases (15.3%) and diseases of the musculoskeletal system and connective tissue (12.2%). Detailed baseline characteristics of study participants are shown in Table 1.

### 3.1. Healthcare Utilisation

The number of hospital admissions and unplanned GP visits were significantly higher in the control group in comparison with the intervention group (*p* = 0.034; *p* < 0.001, respectively). Participants in the control group had 3.35 (95% CI 1.16–10.00) times the risk of hospital admissions and 2.34 (95% CI 1.52–3.57) times the risk of unplanned GP visits compared to participants in the intervention group. No significant difference was found between the groups in the mean number of emergency department visits (*p* = 0.545).

### 3.2. Clinical Outcomes

Within- and between-treatment differences were assessed for the intervention and the control group in all clinical parameters. There was a significant dependent and independent effect of intervention and time on blood pressure, HbA1c, and lipid profile changes. According to the factorial two-way ANOVA, a significant reduction in SBP (*p* = 0.038), DBP (*p* = 0.001), TC (*p* = 0.014), LDL-C (*p* = 0.005), and HbA1c (*p* = 0.045) was observed in the intervention group at 1 year compared to the control group (Table 2).

A significant within-treatment decrease was found in SBP (*p* < 0.001), DBP (*p* < 0.001), and LDL (*p* = 0.021) in patients who received CMM services (pharmacy intervention) (Table 3). SBP decreased by 6.5%, DBP by 6.3%, and LDL-C by 9.2% in the intervention group after 1 year of intervention. At the study baseline, only 50.8% of patients pertaining to the intervention group had controlled hypertension, whereas this figure significantly increased after the pharmacy intervention to 84.6% (*p* < 0.001). The mean absolute BP, heart rate, TC, HDL-C, triglycerides, HbA1c, and fasting blood glucose did not differ significantly between both patient groups at baseline. The LDL-C was the only parameter that differed significantly between both patient groups at baseline, being lower in the intervention group (*p* = 0.015).

### 3.3. Drug Therapy Problems

A total of 317 consultations were carried out in the intervention group, with an average of 4.9 ± 2.6 consultations (mean ± SD) per patient. At the initial assessment, a total of 242 DTPs were identified with an average of 3.8 ± 1.9 (mean ± SD) DTPs per patient. Overall, across all consultations, 563 DTPs were identified. The most prevalent DTPs were “dosage too low” (35.5%), followed by “needs additional therapy” (25.6%) and “dosage too high” (11.9%). Table 4 lists the prevalence of DTP categories. The medications most frequently associated with DTPs were calcium channel blockers (8.3%), statins (7.2%), and beta blockers (6.7%).

## 4. Discussion

To the best of our knowledge, this is the first prospective, open, controlled pre- and postintervention study assessing the clinical impact of the CMM services in older patients with hypertension and established CVDs in Europe and beyond. The obtained results indicate that provision of the pharmaceutical care practice in the primary healthcare setting in Croatia improves patients’ clinical parameters such as blood pressure, TC, LDL-C, and HbA1c, and reduces healthcare utilisation. The results of this study are consistent with previous research, indicating improvements in clinical outcomes and avoidance of healthcare service utilisation in the CMM group [9] and interprofessional collaborative practices in general [40]. Furthermore, this is the first quasi-experimental study with the inclusion of a non-random control group that used the methodology of Cipolle et al. [36] in the provision of pharmaceutical care to older CV patients. A quasi-experimental type of study is widely supported by the WHO [37], as it allows researchers to examine a single question in a “real-world” scenario where true experiments cannot be used for ethical or practical reasons.

Of particular note is that the percentage of patients at blood pressure goal improved remarkably over the course of the study from 50.8% to 84.6%. In congruence with previously published research [24,32,33,40,41], our study findings showed a clinically significant reduction both in SBP (9.0 mmHg) and DBP (4.9 mmHg). As reported by the European Society of Cardiology, meta-analyses of RCTs have shown that a 10 mmHg reduction in SBP or a 5 mmHg reduction in DBP has a strong clinical impact on all major CV events, all-cause mortality, stroke, coronary events, and heart failure, hence rendering our study findings highly relevant [38]. Similarly to our study, Zillich and co-workers found a significant SBP (7.1 mmHg) and DBP (3.2 mmHg) reduction in patients with hypertension over a 1 year period [33], while Prudencio et al. found a significant reduction in SPB (7.4 mmHg), albeit without any change in DBP [34]. However, unlike in the studies conducted in a patient-centred medical home model where pharmacists were able to prescribe and discontinue hypertension medications without direct oversight from the primary care physician [33,41], pharmacists providing direct patient care in our study could not change therapy without primary care physician’s authorisation. A vast array of evidence, including the largest database published until now [36], demonstrated the improvement in the impact of CMM services on blood pressure, yet without including the control group as the limitation [9,19,20,31,42].

The results of this pre- and post-intervention study add to a rather scarce evidence base demonstrating the impact of CMM services on healthcare utilisation, and consequently financial savings [10,28,31,32]. In a study that tested the effectiveness of medication management program in 12 community and hospital pharmacy clinics in Asheville, patients were 50% less likely to have a CV-related ED visit and 55% less likely to have a CV-related hospitalisation at the end of the 6 year period, albeit without the comparison group [31]. Our study is one of the first that looked at the impact of CMM services on medical service avoidance in older CV patients, thus demonstrating significantly more unfavourable outcomes (hospital admissions and unplanned GPs visits) in participants receiving the usual care compared to participants attended by a pharmaceutical care practitioner. Taking into consideration the fact that CVDs are a leading cause of mortality in the world and consequently a major economic burden, by diminishing healthcare utilisation and improving CV risk factors in a general population of patients with hypertension and established CVDs, CMM services could potentially contribute to total healthcare costs savings and prove substantial benefit not only at the primary care level but also at the secondary and tertiary care levels. Further larger-scale research is needed to confirm these findings and to broaden the evidence base with regards to the impact of CMM on healthcare utilisation in older patients with CVDs. Although the economic value of clinical pharmacists in team-based settings is well documented [11], patient access to CMM services in Europe remains limited due to a lack of payer recognition of the value of clinical pharmacists in collaborative care settings and current healthcare payment policy.

The other clinical outcomes, LDL-C and HbA1c, also substantially improved, consistent with findings published elsewhere [19,20,23,24,26,31,34,40]. Moreover, although the LDL baseline values were lower in the intervention group, this study demonstrated a significant improvement in LDL-C compared to the control group. Since patients with diabetes and dyslipidaemia are at increased risk for cardiovascular disease, any improvement in HbA1c and LDL-C, even the slightest, is deemed clinically relevant proving the value of pharmacists’ interventions, that is, CMM services [39,43]. Given the positive findings of the study, this proposed model of patient-centred pharmacist care may offer a viable solution for medication mismanagement in healthcare systems across the world.

In addition, we argue that significantly more prevalent polypharmacy detected in the intervention group could partly be explained by the data collection process. Namely, comprehensive data collection conducted in CMM services undoubtedly resulted in a more detailed medication record, thus contributing to a higher incidence of polypharmacy in patients receiving this service in comparison with the patients receiving usual care. Furthermore, in accordance with previously published work, a higher number of DTPs identified in polymedicated patients was found in the intervention group [10,18].

The most prevalent DTPs identified during CMM visits included “needs additional therapy” and “dosage too low”, as reported elsewhere [10,18]. This emphasises the underutilisation of effective therapy in hypertensive patients with CVDs, adversely impacting both clinical and economic outcomes. The fact that patients are receiving inadequate dosages of medications to provide a therapeutic benefit is frequently encountered in the practice, thus pointing to the ever-greater need for enforcing the offering of CMM services, according to the comprehensiveness of pharmaceutical care practice, for populations with chronic medical conditions.

The current study has several limitations. First, the non-randomisation of the conducted study could have led to the underestimation of the obtained results. However, we argue that this study design was the only ethically acceptable approach, allowing a “control” GP to provide unbiased medical care, hence precluding the Hawthorne effect that could have possibly masked the effect of the intervention. Moreover, a small reduction in HbA1c (0.283%) in the intervention group could be explained by a smaller number of participants with diabetes mellitus. We strongly believe that this reduction could have been clinically more prominent had we included more diabetic patients for a longer study period. Despite all of the study limitations, it is important to emphasise that the results of this study showed the robust statistical and clinical impact of the provided service, even though the study started simultaneously with the process of the early-stage implementation of the service in the Croatian health system. Additionally, it should be noted that the data collection was hindered by the COVID-19 lockdown which reduced post-COVID healthcare accessibility.

## 5. Conclusions

In conclusion, the present study indicates that CMM services can strongly decrease the healthcare utilisation, and significantly improve blood pressure, LDL-C and HbA1c in patients with hypertension and established CVDs at the primary care level. The high prevalence of identified and resolved DTPs in this study confirms the appropriate provision of CMM services in the Croatian healthcare setting and demonstrates how this service can improve the effectiveness of patients’ medications. However, for the service to be fully incorporated into the primary healthcare setting in Croatia, well-prepared and competent pharmacists need to be available in the system. Thus, teaching the practice of pharmaceutical care and CMM services should be made a priority in pharmacy schools. Moreover, further research of the impact of pharmacists’ provision of CMM services on economic outcomes is needed.

## Figures and Tables

**Table 1:** Baseline characteristics of study subjects.

Characteristic	Group		*p*
	Intervention	Control	
Sample size (*n*)	65	68	
Age (years) *	72.4 ± 4.6	73.0 ± 4.7	0.447
Gender female/male	43/22	42/26	0.598
BMI *	29.5 ± 4.9	29.0 ± 4.8	0.584
Alcohol consumption yes/no	15/50	13/55	0.576
Smoking status yes/no	2/63	9/59	0.033 **
Physical activity yes/no	28/37	48/20	0.001 **
Level of education primary/secondary/higher	3/31/29	21/40/7	<0.001 **
Polypharmacy (≥5 medications) yes/no	64/1	48/20	<0.001 **
Type 2 diabetes mellitus yes/no	26/39	17/51	0.064
Hyperlipidaemia yes/no	35/30	33/35	0.400
Medications used per patient at the initial visit *	10.8 ± 3.6	5.8 ± 2.5	<0.001 **
Medications used at the initial visit	699	394	
Diagnoses per patient at the initial visit *	7.9 ± 3.4	8.8 ± 2.5	0.071
Diagnosis at the initial visit	510	598	

BMI, body mass index. * Data expressed as mean ± SD. ** For smoking status, physical activity, level of education, polypharmacy, and number of medications, statistically significant differences between groups were found. Hence, the additional test was conducted to ensure that these parameters did not affect the end-point results. Factorial ANOVA and correlation test showed that the intervention and control group were compatible for comparison, regardless of initial differences (*p* > 0.05).

**Table 2:** Between- and within-treatment change from baseline differences.

Parameter	Control Group Baseline vs. End-Point ^a^	Intervention Group Baseline vs. End-Point ^a^	Baseline Control Group vs. Intervention Group ^a^	End-Point Control Group vs. Intervention Group ^a^
SBP (mmHg)	0.103	0.002 ^b^	0.630	*0.038* ^c^
DBP (mmHg)	0.883	*0.007* ^b^	0.576	*0.001* ^c^
TC-C (mmol/L)	0.934	0.555	0.075	*0.014* ^c^
LDL-C (mmol/L)	0.495	*0.021* ^b^	0.015 ^d^	*0.005* ^c^
HDL-C (mmol/L)	0.347	0.786	0.632	0.471
Triglycerides (mmol/L)	0.113	0.580	0.998	0.325
HbA1c (%)	0.244	0.526	0.839	*0.045* ^c^
FBG (mmol/L)	0.931	0.171	0.420	0.650

^a^ Fisher’s LSD. ^b^ The SBP, DBP, and LDL-C decreased significantly in the intervention group after 1 year. ^c^ A significant reduction in SBP, DBP, TC-C, LDL-C, and HbA1c was observed in the intervention group in comparison with the control group after 1 year. ^d^ A significant baseline difference between groups was found in LDL-C.

**Table 3:** Change in clinical parameters within control and intervention groups.

Parameter	Control Group (N = 68)		Δ (%)	Intervention Group (N = 65)		Δ (%)
	Baseline	End-Point		Baseline	End-Point	
SBP (mmHg)	139.74	135.21	−4.53 (−3.24)	138.39	129.37	−9.02 (−6.52)
DBP (mmHg)	80.79	81.06	0.27 (0.33)	79.78	74.79	−4.99 (−6.25)
TC-C (mmol/L)	4.98	5.00	0.02 (0.40)	4.62 *	4.51 *	−0.11 (−2.38)
LDL-C (mmol/L)	3.02	2.91	−0.11 (−3.64)	2.61 *	2.37 *	−0.24 (−9.20)
HDL-C (mmol/L)	1.37	1.42	0.05 (3.65)	1.39 *	1.38 *	−0.01 (−0.72)
Triglycerides (mmol/L)	1.40	1.76	0.36 (25.71)	1.40 *	1.49 *	0.08 (5.71)
HbA1c (%)	7.25 **	7.71 **	−0.46 (−6.34)	7.16 **	6.90 **	−0.21 (−3.63)
FBG (mmol/L)	8.48 **	8.72 **	−0.24 (−2.83)	8.40 **	7.72 **	−0.68 (−8.10)

Data expressed as mean ± SD. * Missing data for two patients in the intervention group (N = 63). ** Data for patients with type 2 diabetes mellitus (17 patients in the control group and 26 patients in the intervention group).

**Table 4:** The frequency of DTPs by category in the intervention group across all consultations.

DTP Category	*n* (%)
1. Unnecessary drug therapy	32 (5.7)
2. Needs additional drug therapy	144 (25.6)
3. Ineffective drug	40 (7.1)
4. Dosage too low	200 (35.5)
5. Adverse drug reaction	47 (8.4)
6. Dosage too high	67 (11.9)
7. Nonadherence	33 (5.9)
Total	563 (100.0)

## Data Availability

The datasets generated during and/or analysed during the current study are available from the corresponding author upon reasonable request.
